# Supplementary material for: Coral recruits demonstrate thermal resilience
Source: PeerJ. 2024 Nov 12;12:e18273. doi: 10.7717/peerj.18273 (PMC11566514; doi:10.7717/peerj.18273)
Supplement: Supplemental Information 1 [file peerj-12-18273-s001.docx]

# Purebred and hybrid coral juveniles demonstrate thermal resilience: supplementary materials

**Supplementary material S1.** Mass bleaching at Davies Reef, March 2020.


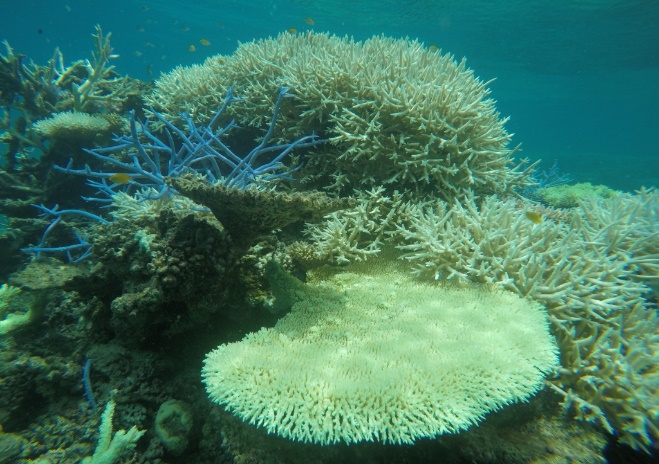

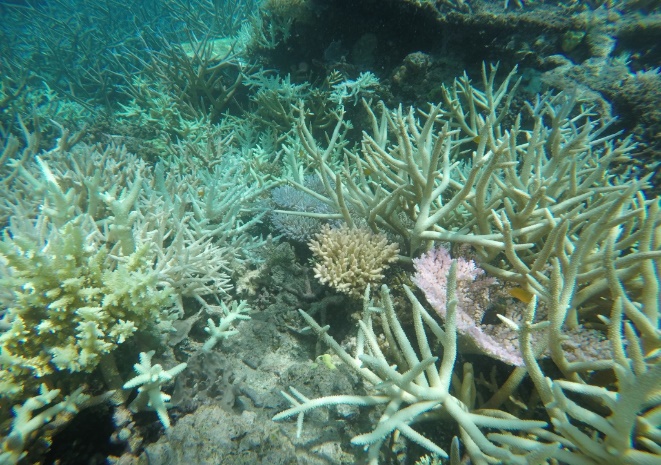

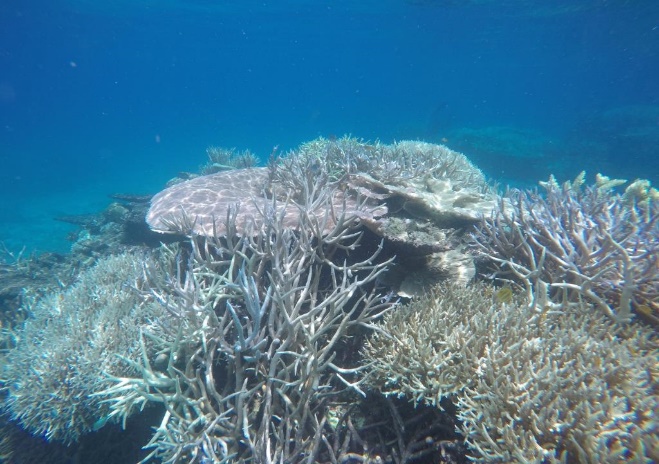

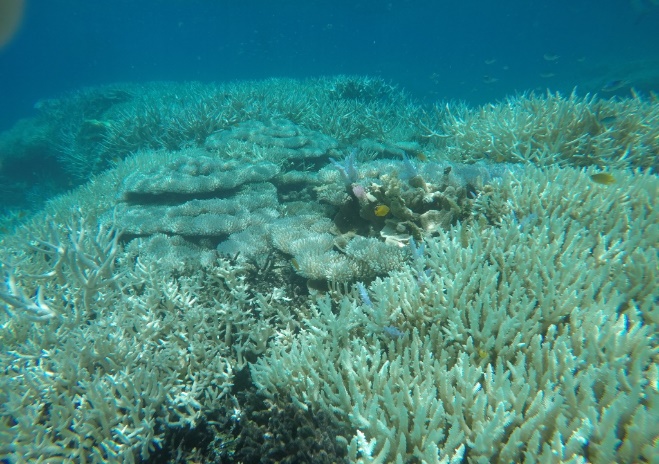


**Figure S1.** Images of mass bleaching at Davies Reef, Great Barrier Reef. Images taken on the 9^th^ of March 2020 by Veronique Mocellin near the Davies Reef Weather Station run by the Australian Institute of Marine Science.

**Supplementary material S2.** Statistical model comparisons

**Table S2.** Table of model AIC values and likelihood ratios and P-values from model comparisons of the statistical models built to test survivorship, size (area in mm2), colouration (as a proxy for bleaching) and F_v_/F_m_ of the coral recruits. The main effects that were tested in each model are included in the table below. An ‘X’ indicates that an interaction term between the two variables framing the ‘X’ was included in the model as well as the main effects of those two variables.

| **Model 1** | **Model 1 AIC** | **Model 2** | **Model 2 AIC** | **Likelihood Ratio** | ***P*** |
| --- | --- | --- | --- | --- | --- |
| Survivorship ~ Offspring Group X Treatment X Time Point | 1103.3 | Survivorship ~ Offspring Group + Treatment + Time Point | 1100.1 | 10.801 | 0.148 |
| Survivorship ~ Offspring Group X Treatment X Time Point | 1103.3 | Survivorship ~ Offspring Group X Treatment + Time Point | 1100.5 | 7.195 | 0.207 |
| Survivorship ~ Offspring Group X Treatment X Time Point | 1103.3 | Survivorship ~ Offspring Group X Time Point + Treatment | 1099.9 | 6.57 | 0.255 |
| Survivorship ~ Offspring Group X Treatment X Time Point | 1103.3 | Survivorship ~ Offspring Group + Treatment X Time Point | 1102.1 | 10.795 | 0.095 |
| Survivorship ~ Offspring Group + Treatment + Time Point | 1100.1 | Survivorship ~ Offspring Group X Treatment + Time Point | 1100.5 | 3.605 | 0.165 |
| Survivorship ~ Offspring Group + Treatment + Time Point | 1100.1 | Survivorship ~ Offspring Group X Time Point + Treatment | 1099.9 | 4.231 | 0.121 |
| Survivorship ~ Offspring Group + Treatment + Time Point | 1100.1 | Survivorship ~ Offspring Group + Treatment X Time Point | 1102.1 | 0.006 | 0.94 |
| Area ~ Offspring Group X Treatment X Time Point | 5845.704 | Area ~ Offspring Group + Treatment + Time Point | 5908.762 | 87.058 | <0.001 |
| Area ~ Offspring Group X Treatment X Time Point | 5845.704 | Area ~ Offspring Group X Treatment + Time Point | 5912.142 | 86.438 | <0.001 |
| Area ~ Offspring Group X Treatment X Time Point | 5845.704 | Area ~ Offspring Group X Time Point + Treatment | 5836.16 | 6.456 | 0.596 |
| Area ~ Offspring Group X Treatment X Time Point | 5845.704 | Area ~ Offspring Group + Treatment X Time Point | 5910.66 | 84.956 | <0.001 |
| Area ~ Offspring Group + Treatment + Time Point | 5908.762 | Area ~ Offspring Group X Treatment + Time Point | 5912.142 | 0.62 | 0.734 |
| Area ~ Offspring Group + Treatment + Time Point | 5908.762 | Area ~ Offspring Group X Time Point + Treatment | 5836.16 | 80.602 | <0.001 |
| Area ~ Offspring Group + Treatment + Time Point | 5908.762 | Area ~ Offspring Group + Treatment X Time Point | 5910.66 | 2.102 | 0.35 |
| Colour ~ Offspring Group X Treatment X Time Point | 2541.524 | Colour ~ Offspring Group + Treatment + Time Point | 2536.737 | 19.214 | 0.084 |
| Colour ~ Offspring Group X Treatment X Time Point | 2541.524 | Colour ~ Offspring Group X Treatment + Time Point | 2537.694 | 16.171 | 0.095 |
| Colour ~ Offspring Group X Treatment X Time Point | 2541.524 | Colour ~ Offspring Group X Time Point + Treatment | 2544.64 | 19.117 | 0.014 |
| Colour ~ Offspring Group X Treatment X Time Point | 2541.524 | Colour ~ Offspring Group + Treatment X Time Point | 2532.771 | 11.248 | 0.339 |
| Colour ~ Offspring Group + Treatment + Time Point | 2536.737 | Colour ~ Offspring Group X Treatment + Time Point | 2537.694 | 3.043 | 0.218 |
| Colour ~ Offspring Group + Treatment + Time Point | 2536.737 | Colour ~ Offspring Group X Time Point + Treatment | 2544.64 | 0.097 | 0.9989 |
| Colour ~ Offspring Group + Treatment + Time Point | 2536.737 | Colour ~ Offspring Group + Treatment X Time Point | 2532.771 | 7.966 | 0.019 |
| F_v_/F_m_ ~ Offspring Group X Treatment X Time Point | -1386.581 | F_v_/F_m_ ~ Offspring Group + Treatment + Time Point | -1380.832 | 19.749 | 0.006 |
| F_v_/F_m_ ~ Offspring Group X Treatment X Time Point | -1386.581 | F_v_/F_m_ ~ Offspring Group X Treatment + Time Point | -1378.429 | 18.152 | 0.003 |
| F_v_/F_m_ ~ Offspring Group X Treatment X Time Point | -1386.581 | F_v_/F_m_ ~ Offspring Group X Time Point + Treatment | -1392.097 | 4.483 | 0.482 |
| F_v_/F_m_ ~ Offspring Group X Treatment X Time Point | -1386.581 | F_v_/F_m_ ~ Offspring Group + Treatment X Time Point | -1379.288 | 19.293 | 0.004 |
| F_v_/F_m_ ~ Offspring Group + Treatment + Time Point | -1380.832 | F_v_/F_m_ ~ Offspring Group X Treatment + Time Point | -1378.429 | 1.597 | 0.45 |
| F_v_/F_m_ ~ Offspring Group + Treatment + Time Point | -1380.832 | F_v_/F_m_ ~ Offspring Group X Time Point + Treatment | -1392.097 | 15.265 | <0.001 |
| F_v_/F_m_ ~ Offspring Group + Treatment + Time Point | -1380.832 | F_v_/F_m_ ~ Offspring Group + Treatment X Time Point | -1379.288 | 0.456 | 0.5 |
